# Nanostructured Plasma Polymerized Fluorocarbon Films for Drop Coating Deposition Raman Spectroscopy (DCDRS) of Liposomes

**DOI:** 10.3390/polym13224023

**Published:** 2021-11-21

**Authors:** Alžbeta Kuižová, Anna Kuzminova, Ondřej Kylián, Eva Kočišová

**Affiliations:** 1 Faculty of Mathematics and Physics, Institute of Physics, Charles University, Ke Karlovu 5, 121 16 Prague 2, Czech Republic; betka.kuizova@gmail.com; 2 Department of Macromolecular Physics, Faculty of Mathematics and Physics, Charles University, V Holešovičkách 2, 180 00 Prague 8, Czech Republic; annakuzminova84@gmail.com

**Keywords:** drop coating deposition Raman spectroscopy, DCDRS, plasma polymers, liposomes, nanostructured surfaces, wettability

## Abstract

Raman spectroscopy is one of the most used biodetection techniques. However, its usability is hampered in the case of low concentrated substances because of the weak intensity of the Raman signal. To overcome this limitation, the use of drop coating deposition Raman spectroscopy (DCDRS), in which the liquid samples are allowed to dry into well-defined patterns where the non-volatile solutes are highly concentrated, is appropriate. This significantly improves the Raman sensitivity when compared to the conventional Raman signal from solution/suspension. As DCDRS performance strongly depends on the wetting properties of substrates, we demonstrate here that the smooth hydrophobic plasma polymerized fluorocarbon films prepared by magnetron sputtering (contact angle 108°) are well-suited for the DCDRS detection of liposomes. Furthermore, it was proved that even better improvement of the Raman signal might be achieved if the plasma polymer surfaces are roughened. In this case, 100% higher intensities of Raman signal are observed in comparison with smooth fluorocarbon films. As it is shown, this effect, which has no influence on the profile of Raman spectra, is connected with the increased hydrophobicity of nanostructured fluorocarbon films. This results in the formation of dried liposomal deposits with smaller diameters and higher preconcentration of liposomes.

## 1. Introduction

The wetting of polymeric surfaces plays a crucial role in different sectors such as the textile industry [1], ink-jet printing [2], metallization of polymers [3], and last but not least in biomedical applications [4,5]. The latter is connected with the fact that the surface wettability governs the interaction of polymers with surrounding media, including the biological suspensions or solutions. This, in turn, influences the attachment, adhesion, or the proliferation of cells or adsorption of biomolecules on polymeric surfaces [6,7,8,9]. In addition, in some situations, the wettability per-se is not the only parameter to be considered. Equally, and in some cases an even more important parameter, is the way in which a liquid drop dries on a surface. As demonstrated in numerous studies, the droplet drying dynamics have a strong impact on the formation of stains after the complete evaporation of droplets that contain non-volatile solutes [10,11,12]. A prominent example of this is the formation of the so-called “coffee-ring”, i.e., the ring-shaped pattern where the edge part comprises accumulated non-volatile solutes [13]. Such structures are highly advantageous, especially for biodetection, as the substances to be detected are highly preconcentrated in well-defined surface locations. One of the biodetection techniques that benefit from the “coffee-ring” formation is the drop coating deposition Raman spectroscopy (DCDRS). In this technique, the Raman signal is acquired from a dried ring pattern where the analyte is highly preconcentrated, which allows the detection and identification of molecules in small volumes (a drop of several µL) and at low concentrations (mM–µM). Furthermore, the great advantage of this approach is the possibility of detecting biological samples at biologically relevant concentrations, which is not possible by conventional Raman spectroscopy (measurement from solutions or suspensions at a standard concentration from cuvette). Previous works showed improvement by 10^3^–10^5^ orders of magnitude as compared to the detection sensitivity of conventional Raman spectroscopy from solutions of proteins [14,15], porphyrins [16] or suspensions of liposomes [17,18]. The DCDRS method was also successfully employed in the detection of dipicolinic acid [19], oligosaccharides [20], food and environmental contaminants [21] and also in a study of real body samples as human tears [22,23,24]. These past studies were concerned about determining the lowest possible detected concentration and finding the spectral fingerprint of protein composition for the tear samples. In addition, the DCDRS technique was shown to be applicable to monitor processes such as albumin glycation [25], catalyzed oxidation [26], and amino acid and peptide phosphate protonation [27] or segregation of impurities and components in mixed solutions [28,29,30]. As for using Raman spectroscopy to study biological samples, it is inevitable to employ a method that can be sensitive even in low concentrations and small volumes. This makes the DCDRS technique an interesting alternative to the more often employed surface-enhanced Raman spectroscopy (SERS) that relies on substrates with precisely designed metal or metal oxide nanostructures (e.g., [31,32,33]).

DCDRS technique proved itself a versatile tool for a broad range of applications, mainly because of its sensitivity to low concentrated samples compared to conventional Raman spectroscopy. Nevertheless, we believe that this sensitivity could be further improved because, despite its simplicity, the applicability of DCDRS is strongly dependent on the used substrate material. It has to be highly hydrophobic to provide as low a diameter of the formed stain as possible to reach the highest preconcentration of the analyte and assure the reliable formation of well-defined rings needed for the DCDRS measurements. For instance, three types of smooth substrates (commercial Teflon-coated stainless steel surface SpecTRIM from Tienta Sciences (Indianapolis, IN, USA), non-commercial polished CaF_2_, and silanized glass surfaces) were tested and compared in a recent study [34]. This comparison revealed significant variation in the Raman signal enhancement in dependence on the wettability of employed surfaces (the hydrophobicity was different because of the different chemical composition/used materials): the highest signal enhancement was observed for fluorocarbon surfaces that exhibited the highest water contact angle. Therefore, we have focused our attention on the fluorocarbon (C:F) surfaces.

The aim of this study is to demonstrate the possibility to enhance further the DCDRS detection capability of fluorocarbon surfaces by their nanostructuring that results in the increase in the surface hydrophobicity. The nanostructured surfaces were used here for the first time since all the previous DCDRS works employed only the smooth ones [14,15,16,17,18,19,20,21,22,23,24,25,26,27,28,29,30]. To meet this general aim, smooth C:F and nanoroughened C:F surfaces with invariant chemical composition were produced using a fully solvent-free plasma-based deposition strategy that combines radio frequency magnetron sputtering of polytetrafluoroethylene target and gas-phase synthesis of Cu nanoparticles. As will be shown, this allows to precisely tailor the roughness and wettability of produced coatings. The DCDRS performance of produced surfaces was subsequently compared using liposome suspension selected as a model biological system.

## 2. Materials and Methods

### 2.1. Fabrication of DCDRS Substrates and Their Characterization

**Figure 1:**
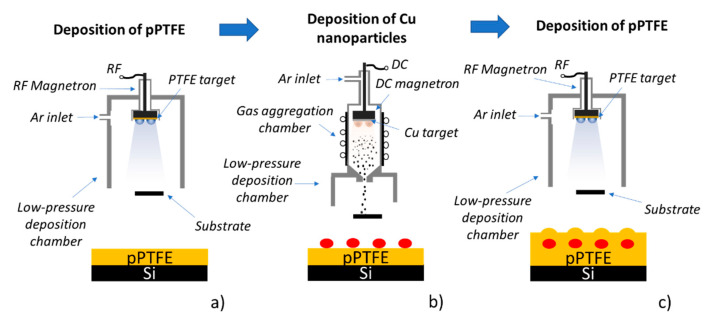
(**a**) Deposition of pPTFE layer. (**b**) Deposition of Cu nanoparticles. (**c**) Overcoating Cu Nps with pPTFE film.

To evaluate the role of surface roughness of fluorocarbon (C:F) thin films on the DCDRS performance, two types of surfaces with identical surface chemical composition were produced—(i) smooth and (ii) roughened. The smooth C:F films were produced by magnetron sputter deposition of 40 nm thick polytetrafluoroethylene (PTFE) layer onto the base platform—one side polished Si wafers (ON Semiconductor, cleaned in ethanol and water) in our case. These substrates are denoted as pPTFE in the subsequent text. The tailor-made roughness of C:F coatings was achieved following a procedure introduced in [35]. This strategy, which is schematically depicted in Figure 1, relies on the additional deposition step, in which the controlled number of nanoparticles (Nps) are sandwiched between two C:F layers (both 20 nm thick in this study). The required surface roughness of resulting coatings is then reached simply by adjusting the size [36] and the number of Nps embedded in the C:F layer [37]. The Cu Nps were deposited in this study utilizing a Haberland-type gas aggregation source (GAS) [38]. It was based on the direct current (DC), water-cooled, 3-inch planar magnetron equipped with a Cu target, which was placed into the water-cooled aggregation chamber (inner diameter of 100 mm) and terminated by a conical output orifice (diameter of 1.5 mm). The GAS was attached to the main deposition chamber, which was pumped by rotary and diffusion pumps. The Cu Nps are produced using Ar as a working gas at the pressure of 40 Pa in the aggregation chamber and using the magnetron current of 400 mA. The deposition time of Cu Nps was 2 and 4 min, and the corresponding samples are denoted as NpsCu2 and NpsCu4, respectively.

The surface morphology of fabricated coatings was determined by means of atomic force microscopy (AFM) and scanning electron microscopy (SEM). The AFM measurements (scanned area 10 μm × 10 μm) were performed using a QuesantQ-Scope 350 AFM. The AFM scans were acquired in the semi-contact mode (scan rate 2 s) using ACLA–10 Si probes (tip radius < 10 nm, AppNano, Mountain View, CA, USA). The AFM images were subsequently analyzed by open-source Gwyddion software. SEM analysis of the produced coatings was done employing the scanning electron microscope JSM 7200F (JEOL, Akišima, Japan). The SEM images were measured in both secondary electron (SE) and back-scattered electron (BE) modes using an accelerating voltage of 15 kV and a working distance of 10 mm. The wettability of produced smooth and nano-roughened fluorocarbon DCDRS substrates was determined by a home-built goniometer. It consisted of a syringe with testing liquid (liposomal suspensions), substrate holder and camera connected to a computer.

### 2.2. DCDRS Measurements

To test and compare the DCDRS performance of produced fluorocarbon-based films, the liposomal suspensions were used. These were prepared from 1,2-dipalmitoyl-sn-glycero-3-phosphocholine (DPPC) powder purchased from Avanti Polar Lipids. After the complete dissolution of lipid in pure chloroform in a glass flask, a stream of nitrogen gas was used to remove the solvent to form a thin layer of lipid spread on a glass surface. Subsequently, deionized water (18 MΩ, Millipore-Q, Darmstadt, Germany) was added to the flask and mixed for lipid hydration and spontaneous vesicles formation. Complete hydration to cloudy liposome suspension was achieved by applying an ultrasonic bath and maintaining the suspension at a temperature of 10–15 °C above the main phase transition of DPPC (41 °C) for about half an hour. Apparatus LiposoFast-BasicTM (Avestin, Inc., Mannheim, Germany) with polycarbonate membrane filter with 100 nm pores was used to obtain a unilamellar suspension. The liposomal suspension was extruded through a membrane filter approximately thirty-five times at a temperature of 50 °C. The detailed standard preparation procedure can be seen elsewhere [39]. The final concentration of liposomal suspension was 1 mg/mL (1.36 mM). This stock suspension was subsequently diluted to 0.5 mg/mL, 0.25 mg/mL and 0.125 mg/mL (0.68 mM, 0.34 mM and 0.17 mM, respectively) and all four concentrations were finally used for DCDRS measurements on smooth and nanostructured fluorocarbon substrates.

Liposome suspensions at all concentrations were deposited on substrates as 2 μL droplets and left to dry at room temperature for about half an hour. All DCDRS spectra from ring patterns were recorded by Raman microspectrometer LabRAM HR800 (Horiba Jobin Yvon, Longjumeau, France) in the back-scattering arrangement. The Microspectrometer was equipped with 300 grooves/mm grating and a nitrogen-cooled charged coupled device (CCD) for collecting the scattered light. Irradiation by He-Ne laser with excitation line at 632.8 nm was employed with final power on samples set to 6.4 mW. All spectra were recorded with 400 µm pinhole diameter, 100 µm entrance slit width and 50× ULWD (ultra-long working distance) objective with the spectral acquisition of 60 × 1 s. White light images of formed, dried rings patterns were taken by using objective 5×. Lateral parameters as widths and diameters of rings deposits were determined from white light images using ImageJ software. The lateral widths of the formed rings were measured at 10 randomly selected positions on the ring and the presented values correspond to the average of these measurements.

### 2.3. Treatment of Measured Spectra by Factor Analysis (FA)

The measured spectra were treated with background corrections and factor analysis by the in-house software developed by J. Palacký [40]. Factor analysis as a multivariate mathematical technique uses a singular value decomposition algorithm for reducing matrices of data to their lowest dimension [41]. The procedure consists of solving the eigenvalue equation that provides orthonormal subspectra S_j_(υ), orthonormal matrix of corresponding scores V_ij_ and a set of singular values W_j_ (weights). A linear combination of computed subspectra can express each original measured spectrum Y_i_(υ) as:

(1) $$Y_{i}\left(\upsilon \right)=\overset{m}{\underset{j=1}{\sum }}W_{j}V_{\mathrm{ij}}S_{j}\left(\upsilon \right)$$

The number *m*, factor dimension, is a minimal number of subspectra that are needed for the best approximation and reconstruction of measured (original) spectra. The output of FA is a set of subspectra, their statistical weights (singular values), residuals errors and normalized coefficients (scores), which indicate the relative presence of subspectra in individual spectra. To analyze measured Raman spectra, the 1st subspectrum is a weighted average of experimental spectra, and all other (second and each subsequent) subspectra reflect various spectral changes. The information from individual subspectra is independent because of their orthogonality. To obtain the factor dimension, singular values assigned to the subspectra can be used, where the significant drop in the value (several orders) is crucial. The number of values before the critical drop indicates the dimension.

## 3. Results

### 3.1. Characterization of C:F Substrates

**Figure 2:**
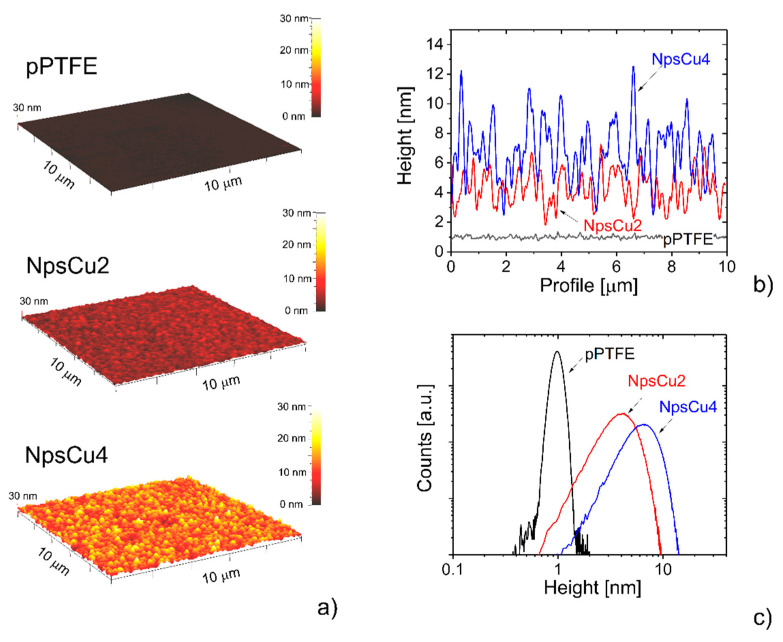
(**a**) AFM images of substrates used for the DCDRS measurements together with their (**b**) height profiles and (**c**) height histograms. The height profiles and height histograms were determined from the AFM images using standard procedures in the program Gwyddion.

The first step of this study was the characterization of fabricated fluorocarbon-based DCDRS platforms. As mentioned, two distinct types of surfaces for the DCDRS measurements were investigated: smooth pPTFE coatings and C:F coatings with nano-roughness induced by the presence of Cu Nps. The corresponding AFM images of these materials are depicted in Figure 2, where the typical height profiles of all samples and their height histograms are plotted, too. As can be seen, the pPTFE is indeed smooth with a root mean square roughness (Rms) of 0.1 nm, while the increasing number of Cu Nps in the coatings results in more nanostructured character of the coatings and in a gradual increase in their roughness with the growing number of Cu Nps in the films. According to the AFM measurements, the values of Rms were found to be 1.3 nm in the case of NpsCu2 sample and 2 nm in the case of sample NpsCu4. These values are comparable with previously reported values for systems with Ag nanoparticles having similar size (diameter 14 nm) deposited for several minutes and overcoated with pPTFE layer (Rms ~ 2 nm for 2 min deposition time of Ag Nps) [38].

**Figure 3:**
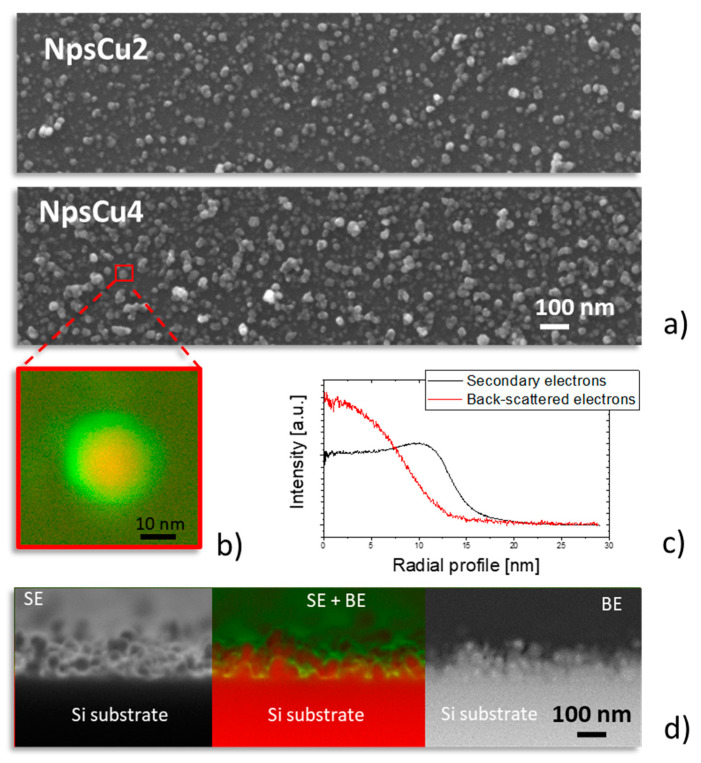
(**a**) Top-view SEM images of samples NpsCu2 and NpsCu4 measured in SE mode. (**b**) Composite SE (false green) and BE (false red) image of detail of surface nanostructure of sample NpsCu4 and (**c**) corresponding radial profiles. (**d**) Cross-section of NpsCu4 coatings measured in SE mode (**left**), BE mode (**right**) and composite SE and BE SEM image (**middle**).

The nanostructured character of the samples that contain Cu Nps was also confirmed by SEM with clearly distinguishable Cu Nps (see Figure 3a). The investigation of surface nanostructures using both SE and BE modes of the scanning electron microscope revealed that the Cu Nps are fully coated by a C:F layer (Figure 3b). According to the measured radial profile of intensity of BE signal (false red) that highlights the material contrast (the higher intensity may be ascribed to the presence of Cu Nps in our case), the size of individual Nps is around 14 nm. Such value corresponds to the value reported in the previous work for the Cu Nps prepared under similar conditions [38]. Furthermore, the thickness of the C:F overcoat deduced for the radial profile of nanostructures visualized in the SE mode (false green), i.e., in the mode that is primarily sensitive to the morphology of the samples, was found to be around 6 nm. This value is markedly lower than the thickness of the C:F overcoat that was 20 nm. Such difference is most likely connected with a partial penetration of pPTFE below the copper Nps due to the favored downhill current of the plasma polymer-forming species from the outmost surface of the Nps down to the substrate or into the inter-particle voids [42].

### 3.2. Wettability and Drying of Liposome Suspension

A model dipalmitoylphosphatidylcholine (DPPC) was chosen to prepare a liposome suspension and subsequently to study the influence of smooth and nano-roughened substrates on the wetting/drying process of deposited samples. 2-μL droplets of DPPC suspension at four concentrations (1, 0.5, 0.25 and 0.125 mg/mL) were deposited on the above-mentioned substrates and left to dry at room temperature.

The first important observation is a significant increase in the initial contact angle value of deposited droplets observed with the change of smooth pPTFE substrate to the C:F substrate with deposited nanoparticles: the static contact angle increased from 108° measured on smooth C:F films to 130° and 135° in the case of NpsCu2 and NpsCu4 samples, respectively. Such changes in surface wettability are in qualitative agreement with Wenzel’s wetting model, which predicts the enhancement of hydrophobicity of hydrophobic materials upon their roughening [43]. This behavior was found to be independent of the concentration of freshly deposited suspension as the differences of the contact angles for the same substrate were always in the range ±3°.

**Figure 4:**
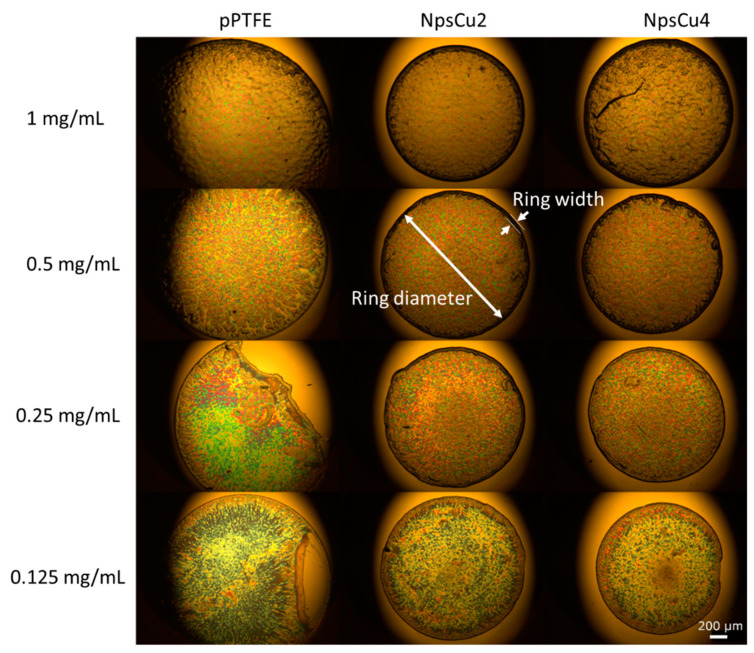
White light images of formed patterns after evaporation of DPPC liposome suspension at four concentrations on three different substrates.

Next, representative white light images of the liposomal deposits after the complete evaporation of the liquid phase of the suspension presented in Figure 4 demonstrate the typical “coffee-ring” formation after an evaporation process for each deposited droplet on each substrate. As for smooth substrate, the evaporation process led to the formation of compact rings only for concentrations of 1 mg/mL and 0.5 mg/mL (as already observed for commercial smooth substrate SpecTRIM (Tienta Sciences, Indianapolis, IN, USA) in the study [15]). A tendency to form a ring in case of lower concentrations could also be observed. However, this had failed, and a pattern with an incomplete ring was created. This is connected with the presence of the constant contact angle (CCA) drying phase during the droplet evaporation [44] in which the triple line of the drying droplet slides on the smooth pPTFE substrate, as shown in our previous study [37]. In contrast, the heterogeneities in the C:F surface topography induced by the presence of Cu Nps lead to the suppression of the CCA drying phase. Thus, the droplet stays pinned on the surface during its evaporation. The droplet pinning, in turn, allows for the formation of complete and well-defined rings after the complete droplet evaporation also for the lower concentrations.

**Figure 5:**
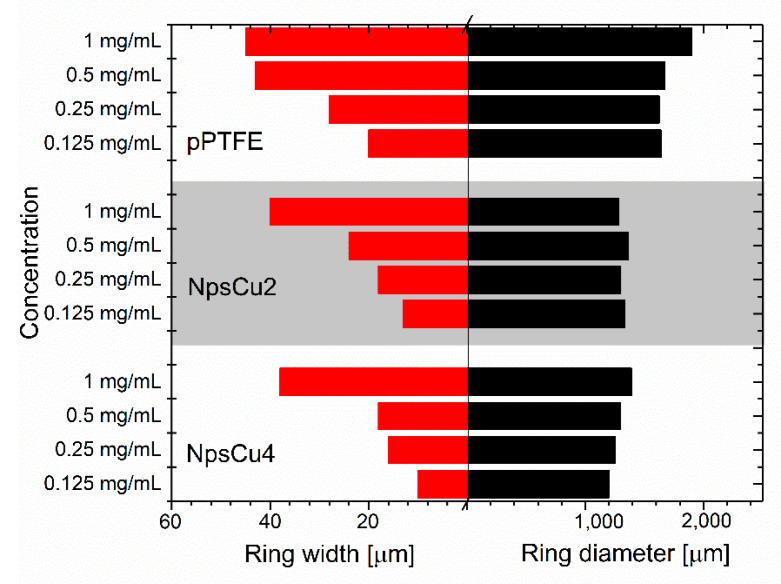
Lateral widths and diameters of the rings formed after the DPPC liposome suspension evaporation.

Another effect visible in Figure 4 and quantitatively summarized in Figure 5 is a decrease in the mean diameters and widths of rings formed after the droplet evaporation. As the hydrophobicity and thus also the contact angle increased, the initial contact radius for the droplets with the same volume had to decrease. Because of this, the diameter of the dried DPPC ring decreased in all cases as the wettability decreased. Furthermore, it was found that the initial concentration of the deposited droplet influenced the final structure of the dried pattern, namely the width of the formed ring: deposited drops of lower concentrations dried out into the patterns with a narrower width of the final ring, as shown in Figure 5. This phenomenon is supposed to be connected with the lower number of liposomes that may form the ring. It was also observed that greater roughening (in the case of NpsCu4) did not cause a more pronounced decrease in the diameters and widths of the formed rings, which is consistent with similar wettability of both samples.

### 3.3. DCDRS Performance

**Figure 6:**
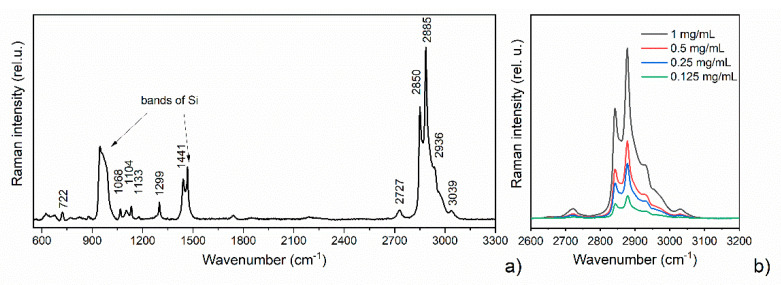
DCDRS spectra of dried liposome suspension from ring-shaped pattern on smooth pPTFE substrate (**a**) at DPPC concentration of 1 mg/mL in the spectral interval 550–3300 cm^−1^. Marked bands of Si originate from the base layer of the substrate; and (**b**) comparison for the upper dominant region at four different concentrations.

Polymeric fluorocarbon smooth surface (pPTFE) was used for testing DCDRS performance of dropped samples of liposomal suspension at different concentrations. For each dried deposit, 25 Raman spectra over the whole ring were accumulated. Acquired spectra for each concentration were treated by baseline adjustment procedure using in-house software to eliminate background signal variations [40]. A typical example of the whole measured spectral interval 550–3300 cm^−1^ for DPPC liposomes by DCDRS on pPTFE substrate is presented in Figure 6a. The upper region 2600–3200 cm^−1^ shown in Figure 6b represents the dominant, more intense part corresponding to the C–H stretching vibrations. Specifically, the spectral band of 2850 cm^−1^ is assigned to the C–H symmetric stretching, 2885 cm^−1^ to the C–H antisymmetric stretching, and the shoulder at 2936 cm^−1^ corresponds to terminal CH_3_ symmetric stretching vibrations. The lower part 550–1800 cm^−1^ is less intense and can be more affected by the background signal during the measurement. The three spectral bands in the 1000–1150 cm^−1^ region are assigned to C–C stretching, the band 1299 cm^−1^ is assigned to the CH_2_ twisting and the signal at 1441 cm^−1^ corresponds to CH_2_ bending [17,45,46]. To compare the signal for different concentrations, the dominant part of spectra sensitive to the phase transitions changes or conceivable interaction with the substrate was used [46]. After the background correction, the averaged spectra for each concentration were compared as shown in Figure 6b, where a decrease of the signal with concentration is clearly demonstrated. This confirms that polymeric fluorocarbon smooth substrate can serve as a suitable hydrophobic DCDRS substrate for liposomal suspension.

**Figure 7:**
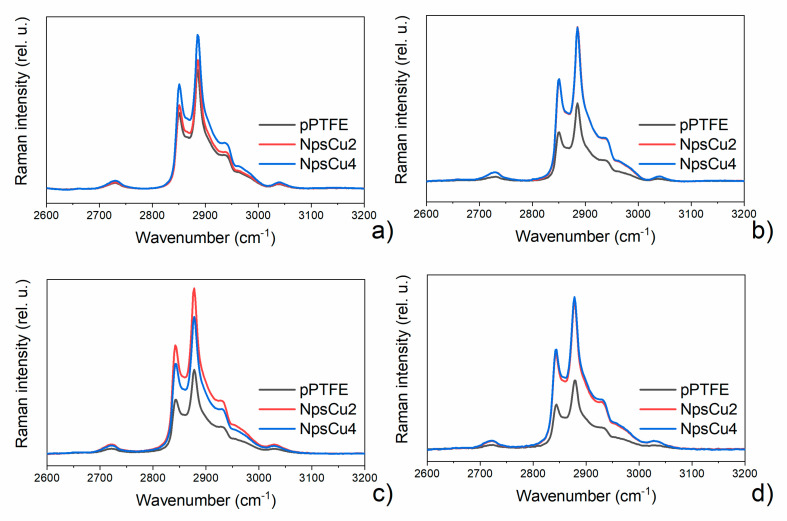
Averaged spectra (baseline corrected) for (**a**) 1 mg/mL, (**b**) 0.5 mg /mL, (**c**) 0.25 mg/mL and (**d**) 0.125 mg/mL deposited concentration of DPPC in the form of liposome suspension.

Afterwards, the DCDRS performance of nano-roughened hydrophobic surfaces (NpsCu2 and NpsCu4) for the same samples of liposomal suspension was studied and compared to the performance of the smooth pPTFE substrate. In total, 5 DCDRS spectra were accumulated similarly as for the pPTFE substrate focusing on the dominant part of spectra 2600–3200 cm^−1^. The baseline-corrected spectra were averaged for each concentration and substrate and compared to each other, as shown in Figure 7. Obtained results showed that the intensity of DPPC spectral bands for each concentration varied in the case of the nanostructured substrates with Cu Nps. For lower concentrations (0.5 mg/mL and 0.125 mg/mL) only a minor intensity difference is present for both nanostructured substrates. At 1 mg/mL, the averaged spectrum from NpsCu4 is more intense than from NpsCu2, but at 0.25 mg/mL the performance from NpsCu2 is better.

**Figure 8:**
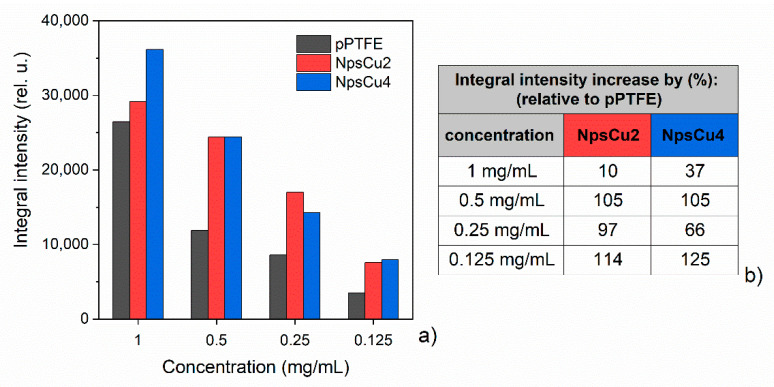
(**a**) Comparison of the integral intensity of averaged DPPC spectra in the region 2780–3020 cm^−1^ for smooth pPTFE and nanorough NpsCu2, NpsCu4 substrates with (**b**) the integral intensity increase (in %) for nanostructured substrates relative to the smooth pPTFE substrate.

To demonstrate the DCDRS performance of smooth versus nanostructured substrates, integral intensities in the region 2780–3020 cm^−1^ were calculated. These values, as well as the increase in intensity (in %), are summarized in Figure 8. The significant about twofold increase in integral intensity for nanostructured NpsCu2 substrate relative to the pPTFE one at a concentration of 0.5, 0.25 and 0.125 mg/mL when increased by 105, 97 and 114%, respectively, is clearly seen. Unlike in the case of the concentration 1 mg/mL this increase is only by 10%. Moreover, these results show that the relevant improvement is achieved when the smooth pPTFE substrate is replaced by the nano-roughened NpsCu2, but the additional increase in deposited nanoparticles (NpsCu4) does not guarantee even better performance. The mechanism is based on a more efficient preconcentration of the liposomes from suspension into the dried ring deposit due to the nano-roughened substrate.

The previous comparison of averaged spectra illustrates only the intensity difference but does not give any information of possible changes in spectral shape due to the side effects, such as an interaction of DPPC with the substrate. A factor analysis (FA) was therefore used as a suitable tool for better determination of the performance for employed hydrophobic substrates. This robust treatment can show (across the different substrates) if any other than intensity changes are present in measured spectral sets. One spectral set for FA consisted of 75 spectra in total, 25 spectra for each substrate.

**Figure 9:**
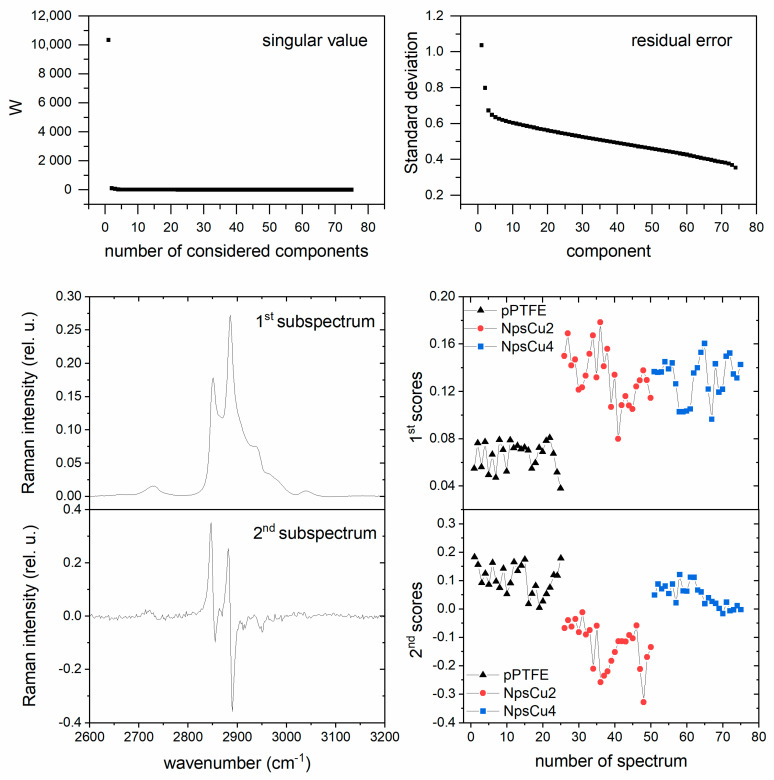
Results of FA for deposited liposome suspension at concentration of 0.5 mg/mL of DPPC, in the particular singular value assigned as W, standard deviation, first and second subspectrum and their belonging scores.

Due to the nature of the studied system, we expected that the factor dimension would be one. In this case, no change in spectral shape caused by phase transition or interaction between DPPC and substrate would be present, and only intensity variations could be observable. The FA results (seen in Figure 9 for an example of FA performed for the concentration of 0.5 mg/mL) showed that the factor dimension is indeed equal to 1 as the singular values (W) representing the statistical weight of the spectral component decreased significantly for the second and higher components. Furthermore, the residual errors do not have any significant drop, which implies that the first subspectrum with its scores is sufficient to describe our original set of spectra, and including the second or higher subspectrum does not lower the overall residual error significantly.

The first subspectrum represents the spectral shape of the DPPC liposome spectrum for the selected spectral interval, and sets of coefficients (scores) belonging to it represent the overall Raman intensity for each baseline-corrected spectrum. From the scores belonging to the first subspectrum concentration, it can be seen that coefficients increase when changing the smooth pPTFE substrate to the nanostructured substrates. As for the second subspectrum, its peculiar shape can be produced by the external effect of calibration due to the laser line shifting during the measurements and by the background signal. Similar results, i.e., the shape of the first and second subspectra, relevant statistical weights and residual errors were also achieved for other studied concentrations. Thus, we can conclude that we observe only changes in the intensities of the Raman signal due to the preconcentration and the interaction of the liposomes with the surface does not occur.

## 4. Conclusions

DCDRS appeared recently to be a highly promising tool for biodetection. The main advantages of this technique are its simplicity and the possibility to analyze samples at biologically relevant concentrations. However, further improvement of the detection capabilities of DCDRS is still needed. While previous studies employed smooth DCDRS substrates, in this study, we propose and investigate for the first time a novel platform for DCDRS measurements based on the nanostructured C:F plasma polymer coatings fabricated by means of the vacuum-based and fully solvent-free procedure. This technique was found to enable the fabrication of C:F films with tailor-made surface properties relevant for the DCDRS (roughness and wettability). In order to highlight the advantages of nanostructuring, the performance of nanostructured C:F films are compared with smooth C:F films with the same surface chemical composition. The main results may be summarized as follows:

- The smooth C:F surfaces deposited by magnetron sputtering were found to be suitable for the DCDRS detection of liposomes. This is due to the hydrophobic character of C:F coatings that forces liquid liposome suspensions to form coffee-ring structures after the complete evaporation of the liquid phase, at least at a higher DPPC concentration.
- As it is shown, the DCDRS measurements might be significantly improved when the fluorocarbon coatings are nanostructured. This led not only to the pinning of the drying droplet that allowed for the formation of well-defined rings also in the case of lower DPPC concentration but also to the lowering the diameter of the resulting rings. The latter, in turn, made it possible to reach a higher concentration of liposomes in the rings and, hence, to get two times higher Raman signal intensity as compared to smooth fluorocarbon films without compromising the profile of recorded Raman spectra.
- These findings may pave the way for the development of cheap and disposable platforms for efficient DCDRS-based biodetection.

## Data Availability

The data presented in this study are available on request from the corresponding authors.
